# Not leaving home: grandmothers and male dispersal in a duolocal human society

**DOI:** 10.1093/beheco/arw053

**Published:** 2016-04-06

**Authors:** Qiao-Qiao He, Jia-Jia Wu, Ting Ji, Yi Tao, Ruth Mace

**Affiliations:** ^a^Theoretical Ecology Group, Key Laboratory of Animal Ecology and Conservation Biology, Institute of Zoology, Chinese Academy of Sciences, Beichen West Road, Chaoyang District, Beijing 100101, PR China and; ^b^Human Evolutionary Ecology Group, Department of Anthropology, UCL, Taviton Street, London WC1H 0BW, UK

**Keywords:** duolocal, grandmother, kin selection, matrilineal, Mosuo, sex-biased investment.

## Abstract

Models suggest that dispersal patterns will influence age- and sex-dependent helping behavior in social species. Duolocal social systems (where neither sex disperses and mating is outside the group) are predicted to be associated with mothers favoring sons over daughters (because the latter are in reproductive competition with each other). Other models predict daughter-biased investment when benefits of wealth to sons are less than daughters. Here, we test whether sex-biased investment is occurring in the duolocal Mosuo of southwestern China. Using demographic and observational data from Mosuo, we show support for both hypotheses, in that 1) males are more likely to disperse from their natal household if their mother dies, but females are not; 2) a large number of brothers increases the likelihood that both females and males disperse, whereas a large number of sisters only increases female dispersal; 3) mothers help daughters reproduce earlier and reduce death risk of daughter’s children, but not sons or sons’ children; 4) data on multiple paternity show that female reproductive success does not suffer from multiple partners, and in males multiple mates are associated with higher reproductive success, indicating that mothers can benefit from investing in their sons’ mating effort; and 5) gift decisions reveal similar kin effects to those shown in the demographic data, with mothers helping adult daughters and adult sons equally, but helping only her daughter’s children, not her son’s children. Mosuo mothers may invest resources for parental investment in their daughters and their offspring, while investing in their sons mating effort.

## Lay Summary

Some models predict that when husband and wife live separately in their own natal household, grandmothers maximize their inclusive fitness by favoring their sons over daughters, as daughters are in reproductive competition with each other. Analyses on the Mosuo living in Southwest China show some support for this argument, with grandmothers helping both sons and daughters but in different ways. Helping sons to stay at home may reduce their workload and help them gain mates.

## INTRODUCTION

Kin selection promotes altruism among related individuals, and more altruistic behavior toward close kin than to distant kin or strangers (Hamilton 1964a, 1964b). However, competition between coresident kin can reduce and even remove altruism toward relatives (Reeve et al. 1998; West et al. 2002; Ji et al. 2013). Demography thus influences age- and sex-dependent patterns of helping in social species (Cant and Johnstone 2008). Some models suggest that in social species with duolocal systems (where neither sex disperses) mothers will favor sons over daughters (because daughters are in reproductive competition with each other) (Johnstone and Cant 2010), whereas other hypotheses predict daughter-biased investment in matrilineal societies, where benefits of wealth to sons may be less than daughters (Holden et al. 2003). Here, we test these 2 hypotheses in the rare duolocal dispersal system of the Mosuo of Southwest China.

Most human societies have patrilocal residence and male-biased wealth inheritance (patrilineal systems), whereas quite a few societies have matrilocal residence and female-biased inheritance (matrilineal systems) (Mace 2013). The matrilineal system is often associated with a lack of high-value, controllable resources (Holden and Mace 2003) and weak marriage bonds (Greene 1978). In most matrilineal societies, males disperse and live with their wife’s relatives, but in a small number of societies, neither sex disperses (known as duolocal residence). The Mosuo (also known as the Na) of Southwestern China is one such rare duolocal matrilineal populations (Walsh 2004) (see Wu et al. 2015, for examples of other such groups in the region). Traditionally, not only Mosuo females but also males stay in their natal household throughout life, and males only visit their partners at night. Children live with their mother and other matrilineal kin.

Mosuo inhabit strips of farmland near and around the shores of Lugu Lake at the border of Sichuan and Yunnan Provinces in Southwestern China, surrounded by steep and forested hills that are not suitable for farming. Mosuo families live in large matrilineal households of 3 generations of brothers and sisters and the matrilineal offspring, and the family farm communally. Mosuo houses are large structures centered around a large grandmother’s room, where the grandchildren also sleep and guests are received (Cai 2001). The grandmother plays a key role in running the household, providing a large portion of the child care as well as helping with farming and feeding the family.

There are several coresident breeding women (mean of 2.14 breeding-age females per household in 2007; Wu et al. 2013), who cooperate with child care, domestic and farm labor, and share all the household resources (Walsh 2004; Wu et al. 2013). Domestic labor (except building work) is mostly done by women. Men are rarely seen in the fields except at planting and harvest time (Yan and Liu 2009). Men do more market trading (Wang and Zhan 2009), and historically, they could hunt and fish and many more may have gone to live in monasteries (Cai 2001), but all our informants and our observation confirm that females work harder than males in both the domestic and agricultural spheres.

In our previous research, we have shown that, in a duolocal household, Mosuo males are allowed to reside with their mothers and sisters, who feed them while they do relatively low levels of household domestic or agricultural labor (Wu et al. 2013). Empirical data on working patterns on the farm show that married Mosuo males do less work than married Mosuo females and married Han males (Wu et al. 2013). In addition, Mosuo males (i.e., brothers and uncles) do not compete for household resources used for reproduction as much as females do (Ji et al. 2013) because their children are elsewhere. This could be why they are tolerated in households without being required to work as hard as females.

In this paper, we examine the implications of the duolocal residence system for the investment of mothers and grandmothers on their sons and daughters. Johnstone and Cant (2010) predict that grandmothers in duolocal systems should prefer their sons over daughters because daughters are in competition with each other; investment in sons incurs less cost from competition for reproductive opportunities than in daughters as his offspring live with their mother (Johnstone and Cant 2010). Postreproductive female killer whales (*Orcinus orca*), which live in duolocal groups (Bigg et al. 1990), lead their sons more than daughters in fishing groups (Brent et al. 2015), providing some support for this view. After the death of their mothers, male killer whales are at high risk of mortality (Foster et al. 2012), suggesting mothers are key to keeping adult sons in the group. However, investments of parents and/or grandparents are predicted to be daughter biased in matrilineal societies when the benefits of wealth to sons are less compared with daughters (Holden et al. 2003). As the reproductive success of Mosuo males are reported to be independent of the characteristics of their natal household, we expected daughter-biased investment from grand/mothers. A former test of this hypothesis in Mosuo has shown that wealth had a larger effect on females’ reproductive success than males (Mattison 2011), and Ji et al. (2013) also found almost no effect of wealth on male fitness.

Here, we focus on kin effects to see which kin are investing in sons or daughters. We measure the role of mothers/grandmothers toward offspring of each sex by examining 4 relevant aspects:

i) We tested whether the death of a mother results in more dispersal by sons or daughters from the natal household. We define dispersal as the event that an adult woman (or man) leaves their natal household to live with a partner, either to live in the partner’s natal household (virilocal [or uxorilocal] residence) or to establish their own neolocal nuclear household where they will live with her/his partner and children. Mothers could benefit their inclusive fitness by helping their sons stay at home because sons could mate more freely when not coresident with a spouse; a son does not compete as much for limited reproductive resource in the household as his sister because his children are elsewhere (living with their mother in a different household). We predict that a mother helps keep an adult son in the natal household, whereas his sisters are less keen to do so, as they are less related to him than is his mother, due to the lower relatedness generated by the risk that they may not share a father. Moreover, coresident brothers may divert household resources away from reproduction for sisters and may even become a burden to them.

ii) We examined the implications of the death of a mother/grandmother on the reproductive success of her sons and daughters. A mother helps adult daughters reproduce if Holden et al.’s (2003) hypothesis is supported, but that she helps adult sons reproduce if the alternative hypothesis is supported (Johnstone and Cant 2010).

iii) We presented data on multiple paternity to explain to what degree siblings are likely to be related and whether multiple partners are a cost or benefit to men and women. These results could help reveal evolutionary trade-offs (which close kin to invest in) that a Mosuo woman or man would face in daily life (i and ii) or in an economic game (see iv). Moreover, the latter could show whether a mother could benefit from investing in the mating effort of sons or daughters. Exact estimates of relatedness between siblings in matrilineal societies are hard to come by, although there is a general trend that marriage bonds are relative weak (Schneider and Gough 1961). Rates of multiple paternity in matrilineal societies have been of interest to evolutionary anthropologists as some theories of matriliny hinge on high levels of polygamy as a key variable; some models consider multiple paternity or paternity uncertainty as key (Greene 1978; Hartung 1985; Holden et al. 2003; Fortunato 2012; Rogers 2013; Wu et al. 2013), whereas others consider that the relationship with the returns on or efficiency of investment also needs to be taken into account (Holden et al. 2003; Fortunato 2012; Wu et al. 2013). Archetti (2013) and Wu et al (2013) argue that the major determinant of matriliny or communally breeding females is the degree of resource depletion through division rather than multiple paternity. In the Mosuo, coresident spouses were rare in favor of the more flexible “walking marriages” or “sese” (where partners live apart); such relationships can endure for short or long periods of time (Shih 2009). Early ethnographic reports on the Mosuo by Chinese researchers in the 1950s emphasized this point, but some of the reporting data on multiple male partners may have been exaggerated (Zhan et al. 1980; Yan and Song 1983; Cai 2001). In our first survey of the Mosuo in 2007, duolocal residence was very common, but very low rates of multiple paternities were reported by mothers. However, it should be noted that since the 1980s Chinese government family planning policy has controlled fertility to 3 offspring, and has made illegal the birth of babies outside of marriage, which is likely to have reduced both actual and reported number of reproductive partners per woman and increased the importance of marriage. Furthermore, a trend in favor of more nuclear families may be occurring as the Mosuo system begins to conform with the majority Chinese norm of monogamous marriage (Mattison 2010; Mattison et al. 2014; Ji et al. 2016). Here, we use data from interviews with those over 50 years, whom we asked to report on who was the father of each of their matrilineal siblings. We felt this method would also be more likely to accurately reflect the Mosuo social system among those who would have largely completed their fertility before the family planning policy was enforced in this area in 1982.

iv) We examined to whom women and men chose to give a small monetary gift, as part of an economic game. According to Johnstone and Cant (2010), a mother should give more gifts to sons than daughters, whereas Holden et al. (2003) predicts the opposite.

## METHODS

All procedures described were reviewed and approved by the Animal and Medical Ethical Committee of the Institute of Zoology, Chinese Academy of Sciences, and the Research Ethics Committee at University College of London.

Our analyses are based on data from 2 demographic surveys, a sibling paternity survey, and a gift decision in a Mosuo population of southwest China. In 2007, we conducted a demographic census of 7034 people in all 5 Mosuo administrative villages in Lugu lake Town on the shores of Lugu Lake in the Tibetan borderlands of Sichuan province, China. In 2012, we conducted a second demographic census of this population, along with a birth history and sibling paternity survey for women and men over 50 years. Mortality is now very low in this area. All demographic analyses are based on a subsample of 6919 Mosuo people. From June to July 2013, we conducted a gift game in this population, as a part of a series of economic games.

For each household, 1 adult representative was interviewed about the personal information of all male and female family members as well as household information, which included name, ethnic group, gender, year of birth, animal sign, education, parents’ name, parents’ year of birth, parents’ year of death, year of moving households, occupation, marriage status, residence type, partners’ names, children’s name, children’s year of birth, children’s year of death, children’s gender, and place of residence, GPS location, year of household established, land size, number of livestock, and number of hotels and shops. Normally, representatives could give most answers by themselves. When they could not remember some of the dates or other information, we allowed them to discuss with other members in the household. Even so, sometimes certain questions may be still not properly answered, especially those about the dates of dispersal of people who had moved out of the area for many years. Hence, we excluded those people in the analysis of kin effects on dispersal (223 people in total). We might underestimate men’s partners or children number if a male has a partner/child not known to other household members living outside of the town.

We also interviewed women and men above 50 years for their birth histories and their sibling information; birth history information included time of residence changes, time of start and end of relationship, children’s name, children’s year of birth, children’s year of death, children’s gender, children’s father/mother’s name; sibling information included sibling’s name, sibling’s year of birth, sibling’s year of death, sibling’s animal sign, sibling’s father’s, or mother’s name. Because this dataset relates to the past, we do not have data on wealth or any other socioeconomic covariates for this older generation.

We conducted a series of economic game experiments in the Lugu Lake Town, with a gift decision at the end of the game session. Each game session was played in a group of 20 participants who were gathered together directly before the game from each village (roughly half of whom were women in each group). Participants could see all the other 19 people in the group before the whole session started, but they made decisions one by one in a private room. In the gift decision, each participant received 15 yuan to give to up to 3 different persons. This is a small monetary gift, as 1 day’s wage is around 60–70 yuan in this area. Receivers could be any adult named by the participant who was living in the study site. At the beginning of the gift game, we informed participants that gifts were anonymous, and all gifts from different givers would be summed up and delivered by us to receivers within 2 weeks. Three hundred and twenty-one Mosuo women and 246 Mosuo men aged 18 years and over participated and gave gifts to 1086 receivers in total (only 19.8% of receivers were people who were participants in the same experiment group with givers), in which gifts given to 331 close kin by 232 women, and 159 by 123 men (close kin here mean parents, siblings, children, grandchildren, and nephews and nieces).

### Statistics

We used event history analysis (EHA) to analyze the effect of kin on dispersal from the natal household, age at first birth, and child survival in the first 6 years. EHA is ideal for studying the probability of an event happening over time, as it allows for censoring cases and time-varying explanatory variables (Allison 1984). For the analyses of kin effects on dispersal and age at first birth, discrete-time EHA complementary log–log regressions (Singer and Willett 2003) were done using Stata (v 12.0), from which 2 figures were derived. Mortality events in the first 6 years were rare. Thus, in order to minimize biases, relogit regression (King and Zeng 2001a, 2001b; Choirat et al. 2016) was performed using zelig packages in R (v 3.2.3). Other figures and statistic tests (Mann–Whitney *U* test and chi-square test) were done using IBM SPSS (v 18.0, SPSS Inc).

## RESULTS

### Kin effects on dispersal

**Figure 1:**
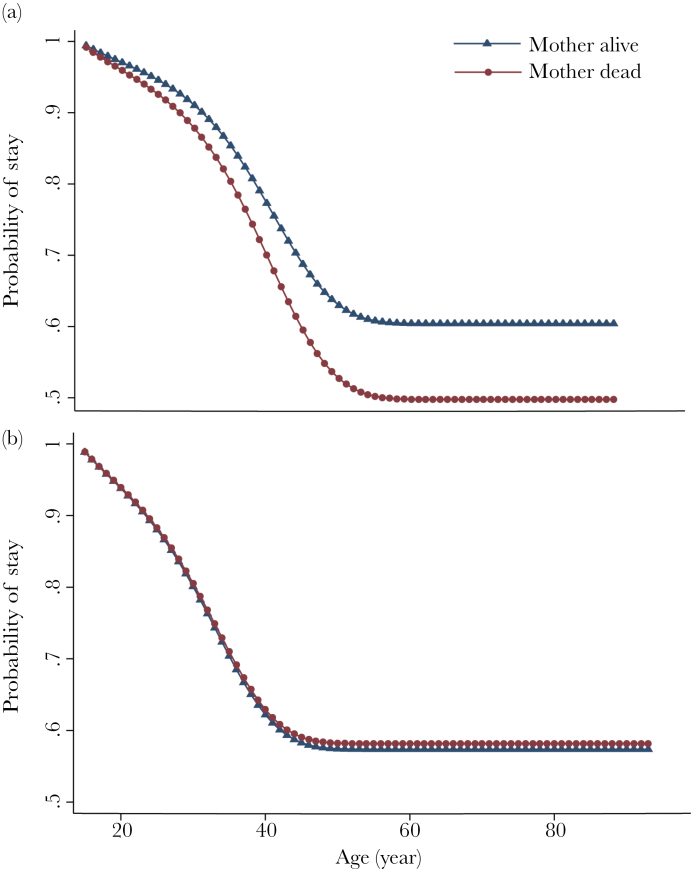
Survival curves derived from a complementary log–log regression (see Table 1 for the full model) for dispersal of (a) male (*n* = 1651) and (b) female (*n* = 1544) offspring experiencing their mother’s death (line with circle)/not (line with triangle).

To examine whether mothers favor their sons by enabling them to stay at home, we examined how the death of mother affected dispersal of sons and daughters. This also allows testing whether reproductive conflict between siblings influenced patterns of dispersal of daughters. Mother’s death significantly increases the hazard of son’s dispersal by 1.4 times but does not affect dispersal by daughters (Figure 1 and Table 1). Father’s death is not associated with the dispersal of sons or daughters (Table 1). The latter finding is in line with other findings that fathers are rarely an important contributor to duolocal households. It seems that the presence of the mother helps keep sons in the household. We also found that the more siblings a Mosuo man’s partner had, the less likely he would disperse into her maternal household (Supplementary Table S1). By keeping sons in the maternal household, mothers may allow them to avoid competition with his partner’s siblings for their limited household resources used for living and reproducing.

**Table 1:** Results of EHA on risk of dispersal (*n* = 29927 person-years for 1544 adult women aged 15 years and over and 31350 for 1651 adult men aged 15 years and over; 375 events for women, and 264 for men)

	Female			Male		
Dispersal	Estimate (SE)	Hazard ratio	*P*	Estimate (SE)	Hazard ratio	*P*
Mother (alive as references category)						
Dead	−0.019 (0.158)	0.982	0.906	**0.332 (0.146**)	**1.393**	**0.023**
Father (alive as references category)						
Dead	0.142 (0.125)	1.153	0.254	−0.042 (0.14)	0.959	0.763
Adult brother number	**0.102 (0.04**)	**1.107**	**0.01**	**0.094 (0.048**)	**1.099**	**0.048**
Adult sister number	**0.119 (0.037**)	**1.127**	**0.001**	−0.010 (0.047)	0.99	0.823
Birth cohort (<1940 as references category)						
1940	0.255 (0.388)	1.290	0.511	0.682 (0.366)	1.977	0.062
1950	0.441 (0.329)	1.554	0.180	0.41 (0.354)	1.507	0.247
1960	**0.698 (0.309**)	**2.010**	**0.024**	0.663 (0.342)	1.941	0.052
1970	**0.902 (0.308**)	**2.464**	**0.003**	0.473 (0.35)	1.605	0.177
1980–1997	**0.937 (0.325**)	**2.553**	**0.004**	−0.626 (0.466)	0.535	0.179
Constant	−**14.811 (1.305**)	**0**	**<0.001**	−**15.117 (1.906**)	**0**	**<0.001**

We used complementary log–log regression with dispersal (dispersed = 1, stay = 0) as dependent variable. The predictors used in the model are mother dead; father dead as time-varying variables; and adult brother number, adult sister number, and birth cohort as time-invariant variables. Sibling numbers only counted those from the same mother, as it is usually mother’s children who live in the same household; sibling numbers were time-invariant variables, due to timing of leaving home (if ever) not being known for everyone. Significant effects are indicated in bold. SE, standard error.

Siblings also affect the dispersal of the Mosuo. The more adult sisters they have, the more likely females but not males are to disperse, implying competition between females; this is likely to be reproductive competition as only females use household resources to raise children. Having many adult brothers increase the likelihood of both female and male dispersals (Table 1). These results suggest that too many adult males are a burden for a household. Although a large number of sisters does not drive brothers out of the household, they do not show any positive effect on keeping them in the household either. This implies that females do not compete with their brothers as much as they do with their sisters, but may be less keen to help them stay than is their mother. These results on dispersal are consistent with previous findings based on reproductive success (Ji et al. 2013) that sisters compete with each other more than with brothers.

### Kin effects on reproductive successes

We examined whether reproductive success was also affected by maternal survival or by other close kin such as siblings. Only 16 mothers and 59 fathers died within 6 years after a child was born (*n* = 3994), and none of the offspring died after the maternal/paternal death. Thus, we did not include parental death in this analysis. Table 2 shows the results of the analysis of other kin effects on children’s survival during the first 6 years, with maternal age controlled. Maternal grandmothers helped grandchildren live through their first 6 years, with the odds of mortality increasing 1.77 times after the death of maternal grandmother (no sex differences). But we found no measurable effect of paternal grandmothers’ death on the survival of their grandchildren during the first 6 years (Supplementary Table S2) nor of grand/mothers’ death on their adult sons’ death (Supplementary Table S3).

**Table 2:** Results of EHA of child survival during first 6 years (*n* = 22941 person-years for 2008 males and 1986 females; 40 events of mortality for males and 48 for females)

Mortality in the first 6 years	Estimate (SE)	Odds ratio	*P*
Sex (male as references category)			
Female	0.181 (0.216)	1.198	0.401
Matri-grandmother (alive as references category)			
Dead	**0.571 (0.262**)	**1.77**	**0.029**
Mother’s age at birth (<21 as references category)			
21–25	−0.473 (0.287)	0.623	0.099
26–30	−**0.827 (0.344**)	**0.437**	**0.016**
31–35	−**0.805 (0.428**)	**0.447**	**0.06**
36+	−0.035 (0.381)	0.966	0.927
Birth cohort (<1950 as references category)			
1950	−0.959 (0.594)	0.383	0.107
1960	−**1.358 (0.525**)	**0.257**	**0.01**
1970	−**1.491 (0.513**)	**0.225**	**0.004**
1980	−**1.57 (0.504**)	**0.208**	**0.002**
1990–2012	−**2.464 (0.524**)	**0.085**	**<0.001**
Constant	−**2.348 (0.525**)	**0.096**	**<0.001**

We used relogit regression for analysis (King and Zeng 2001a, 2001b), with mortality in the first 6 years of child (dead = 1, alive = 0) as dependent variable. The predictors used in the model are matrilineal grandmother dead as time-varying variables, and sex, birth cohort, and maternal age cohort as time-invariant variables. Significant effects are indicated in bold. SE, standard error.

**Figure 2:**
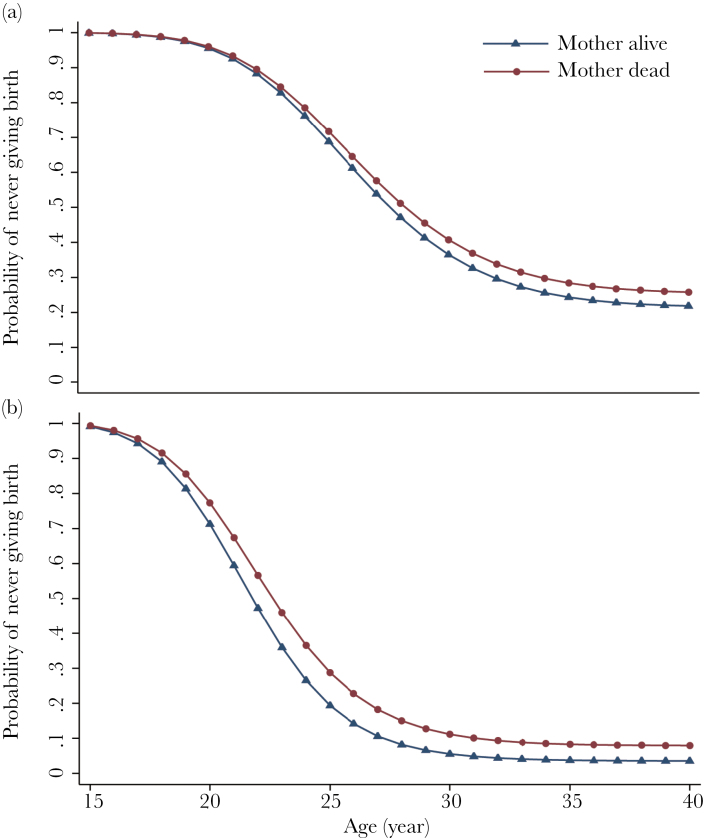
Survival curves derived from a complementary log–log regression (see Table 3 for the full model) for fist birth of (a) male (*n* = 1665) and (b) female (*n* = 1537) offspring experiencing their mother’s death (line with circle)/not (line with triangle). Hazard ratio became 0 after age 40, thus we restrict the *x* axis to age 15–40.

We also analyzed the effects of close kin on the individuals’ age of first birth. We used age at first birth instead of living offspring number as a measure of reproductive successes because, since the 1980s, a Mosuo woman is restricted to only 3 children in her lifetime due to government family planning policy (thus living offspring number may not be informative). Mother’s death and a large number of adult sisters significantly delayed adult women’s age at first birth, for example, the probability of women’s first birth reduced 24.1% after maternal death (Figure 2 and Table 3). Women who ever had an occupation other than being a farmer also had their first birth later (Table 3). Although mother’s death was associated with a slight delay in male age at first birth, none of above variables (parental deaths, sibling number, or occupation) had significant effects on adult male’s age at first reproduction.

**Table 3:** Results of EHA of age at first birth (*n* = 12912 person-years for 1537 adult females aged 15 years and over, and 19360 person-years for 1665 adult males aged 15 years and over; events = 1105 for females and 863 for males)

Age at first birth	Female			Male		
	Estimate (SE)	Hazard ratio	*P*	Estimate (SE)	Hazard ratio	*P*
Mother (alive as references category)						
Dead	−**0.276 (0.119**)	**0.759**	**0.02**	−0.115 (0.11)	0.891	0.294
Father (alive as references category)						
Dead	−0.078 (0.089)	0.925	0.383	0.044 (0.087)	1.045	0.611
Adult sister number	−**0.054 (0.025**)	**0.948**	**0.030**	−0.006 (0.026)	0.994	0.804
Adult brother number	−0.039 (0.024)	0.962	0.114	0.029 (0.027)	1.029	0.287
Birth cohort (<1940 as references category)						
1940	**0.722 (0.179**)	**2.058**	**<0.001**	0.432 (0.215)	1.541	0.804
1950	**0.701 (0.159**)	**2.015**	**<0.001**	0.4 (0.202)	1.492	0.287
1960	**0.53 (0.148**)	**1.698**	**<0.001**	**0.506 (0.191**)	**1.658**	**0.008**
1970	**0.349 (0.146**)	**1.418**	**0.017**	−0.074 (0.189)	0.929	0.696
1980–1997	−0.054 (0.152)	0.947	0.721	−**0.774 (0.201**)	**0.461**	**<0.001**
Occupation (none as references category)						
Ever had one	−**0.592 (0.115**)	**0.553**	**<0.001**	0.061 (0.071)	1.063	0.391
Constant	−**28.647 (1.328**)	**0**	**<0.001**	−**30.343 (1.462**)	**0**	**<0.001**

We used complementary log–log regression for men and women separately, with first birth (one has given birth = 1, not given = 0) as dependent variable. The predictors used in the model are mother dead and father dead as time-varying variables, and adult sister number, adult brother number, birth cohort, and occupation as time-invariant variables. Significant effects are indicated in bold. SE, standard error.

### Polygamy and reproductive success

We presented data on multiple paternities in the Mosuo, so as to clarify whether mother can benefit from investing in sons’ mating effort. Moreover, this measure predicts the extent to which females are less related to their brothers than are their mothers, which may give support to the view that sisters are less willing to help their brothers stay in natal household than is their mother. Relatedness between siblings also allows us to predict to which close kin Mosuo men and women should give gifts in an economic game (see Gift decisions for more details).

**Figure 3:**
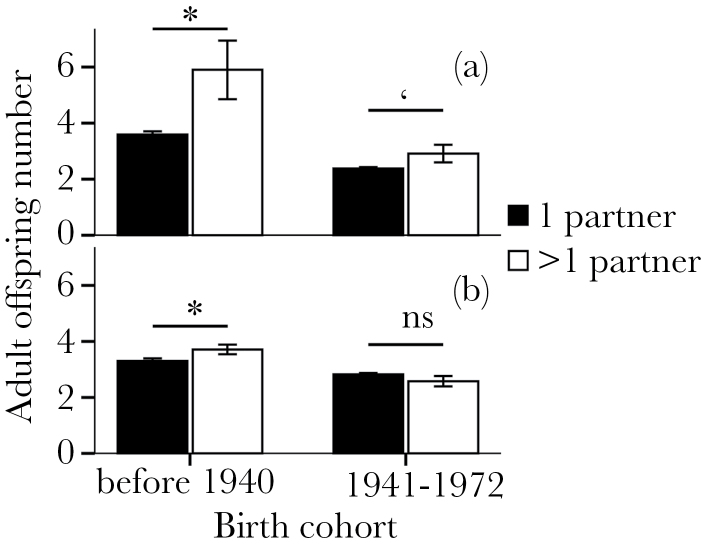
Effects of birth cohort and partner number on adult offspring number of Mosuo (a) males (*n* = 1222) and (b) females (*n* = 1474). Error bars indicate standard error of the mean. Black bars, ≤1 partner; white bars, >1 partner. *P < 0.05; ‘0.1 > *P* ≥ 0.05; ns *P* ≥ 0.1. The reproductive success of males with more than 1 partners was larger than those with one, with the effects significant for men before 1940 (*n* = 405 males, Mann–Whitney *U* = 1099.5, *z* = −2.422, *P* = 0.015) and marginally significant for those after 1940 (*n* = 817 males, Mann–Whitney *U* = 10996, *z* = −1.776, *P* = 0.076). Females who had more than 1 partner had slightly more adult offspring than those who had one before 1940 (*n* = 628 females, Mann–Whitney *U* = 17711, *z* = −2.814, *P* = 0.005) and were not significantly different in the later period (*n* = 846 females, Mann–Whitney *U* = 16184, *z* = −1.207, *P* = 0.227).

We analyzed the relationship between partner number and reproductive successes for Mosuo women and men aged over 40 years. We considered 2 age groups: those individuals born prior to 1941 and those born between year 1941 and 1972. Those in the first group had almost all finished reproduction before the government family planning policy that limits births and requires parents of offspring to be married (which started around year 1982 in this area), and those in the second group had mostly finished reproduction by year 2012 (the year of the latest survey), who were reproducing in the period when births were limited to 3 per woman. Females in the first group had slightly more adult offspring if they had more than 1 partner (*n* = 628 females, Figure 3). Women with only one partner had on average 3.3 children (standard deviation [SD] = 2.154, *n* = 548), whereas those with more partners had on average 3.71 children (SD = 1.552, *n* = 80). There were more offspring for males who had more than 1 partner, and the effect was significant for men in the first age group and marginally significant for those in the second group (*n* = 405 males before 1940 and *n* = 817 males in second period, Figure 3). Men with only 1 partner in the first group had on average 3.59 (SD = 2.338, *n* = 395) children (and 2.38 [SD = 1.453, *n* = 783] for those in the second group), whereas those with more partners had on average 5.9 (SD = 3.315, *n* = 10) children (and 2.91 [SD = 1.832, *n* = 34] for second group). The data for the older generation were less informative for men than women, due to the lower number of reliable reports. Overall, it appears that female reproductive success does not suffer from multiple paternities over their reproductive lives, and multiple partners are associated with an increase in male reproductive success.

We interviewed males and females over 50 years old about the paternity of their siblings. In our first demographic survey, mothers reported that over 95% of offspring were from the same husband, but both behavior and reporting of behavior may have been altered by the family planning policy, the now legal requirement to marry if a child is to be born, and the general spread of a monogamous ethos, from interactions with other groups. The parents of those over 50 years reproduced prior to most family planning policies. However, they lived through a period of social disruption (which included the Great Leap Forward, collectivized farming and the Cultural Revolution that lead to hunger and some starvation across the nation), and a short period in the 1960s when an attempt to impose monogamous nuclear families was briefly enforced (Shih and Jenike 2002). We assumed that reports for sibling paternity should be a more accurate reflection of polyandry in women who completed their fertility prior to the government family policy interventions of 1982 (furthermore in the older generation sibling reports could be the only possible source of information on families from that era because many of the mothers are now dead). However, it cannot be assumed that these data were not also influenced to some extent by the trend toward negative associations with reporting polyandry.

In total, we had 379 datasets containing paternity information on one’s maternal siblings, and these gave us offspring’s paternity information of 288 mothers (Supplementary Figure S1). Extrapair paternity was determined by sibling accounts so there were often multiple informants and a birth was considered extrapair if any sibling said their siblings’ father was different from their own or if a living mother reported this. 20.2% of mothers had more than 1 reproductive partner in their life time (all but 8 of them born before year 1940), plus an additional 3.1% of mothers had only one named partner, but had at least 1 child whose father’s name was unknown to the other siblings, implying that the mother had more than 1 partner (Supplementary Figure S1). Supplementary Table S2 gives the number of siblings with the same mother, full sibling number, and half sibling numbers. Polyandrous reproduction in Mosuo people is not as high as some historical accounts have suggested (Cai 2001), but it is in line with figures estimated for extrapair mating in other matrilineal groups such as the Himba (Scelza 2011). Hence, siblings are somewhat less related to each other on average than their mother is related to them (Supplementary Table S4).

### Gift decisions

We looked at how Mosuo people invested money in their close kin in a gift game, as a test of the pattern of their investment to close kin. In total, 331 close kin received gifts from 232 women and 159 from 123 men participants. Table 4 shows that women and men all give significantly more gifts to their mother than their father. Women give significantly more gifts to sisters than brothers, and more gifts to daughter’s children than son’s children. Men showed rather similar patterns, with gifts strongly focused on female members of their natal family. They also give more gifts to sisters than brothers, but very little to any grandchildren (only 2 gifts both going to daughter’s children). The results from the gift decisions correspond with the patterns of cooperation and conflict between grand/mothers and grand/fathers and sisters and brothers as revealed in the demographic analyses. However, although the demographic data suggests that sisters compete for reproductive resources with other sisters more than with brothers, sisters are more likely to give gifts to each other than to brothers in the gift decision. Although both Mosuo men and women focused on female close kin rather than male close kin, they all gave more gifts to distant kin/non-kin of the same sex as themselves than to those of the opposite sex (Table 4).

**Table 4:** The pattern of gifts given to close kin and others by Mosuo women and men (results of chi-square test, with significant sex differences in number of gifts received indicated in bold)

	Receiver	Male	Female	Chi square	*P*
Female giver	Father/mother	15	75	**40**	**<0.001**
	Brother/sister	42	63	**4.2**	**0.04**
	Son/daughter	46	50	0.167	0.683
	Brother’s/sister’s children	4	22	**12.462**	**<0.001**
	Son’s/daughter’s children	1	13	**10.286**	**0.001**
	Other males/females	95	220	**49.603**	**<0.001**
Male giver	Father/mother	10	38	**16.333**	**<0.001**
	Brother/sister	19	35	**4.741**	**0.029**
	Son/daughter	15	20	0.741	0.398
	Brother’s/sister’s children	3	17	**9.8**	**0.002**
	Son’s/daughter’s children	0	2	n/a	n/a
	Other males/females	172	109	**14.125**	**<0.001**

n/a, not applicable.

We also tested whether Mosuo men or women gave more gifts to mothers (or others). When we only included close kin, the percentage of gifts to mothers was almost the same for men and women (23.9% for men and 22.6% for women). Then, we considered the total number of gifts (646 gifts given by women and 440 by men in total). Women were a little more likely than men to give gifts to mothers, but the effect is not significant (chi square = 2.482, *P* = 0.115, Supplementary Table S5). Mosuo women were more likely than men to give gifts to their own children (chi square = 6.801, *P* = 0.009 for sons; chi square = 4.429, *P* = 0.035 for daughters, Supplementary Table S5). They were also more likely to give gifts to their daughter’s children than men (chi square = 4.663, *P* = 0.031, Supplementary Table S5). There are almost no gifts given to son’s children. Moreover, Mosuo women were more likely than men to give gifts to their close kin rather than to others (chi square = 24.107, *P* < 0.001). For women, 51.2% of gifts were given to close kin, whereas for men the proportion is only 36.1% (Supplementary Figure S2). Mosuo men and women only gave a small percentage of gifts to their spouses (6.6% for men and 3.9% for women), with men giving slightly more gifts to spouses than women (chi square = 4.101, *P* = 0.043, Supplementary Table S5 and Figure S2). Mosuo men were more likely than women to give gifts to other non-kin (i.e., non-kin except spouse or affines; chi square = 34.843, *P* < 0.001, Supplementary Figure S2), suggesting that men focused on social relationships with non-kin more than women.

## DISCUSSION

Our earlier work found that the reproductive success of males appears to be almost entirely dependent on the characteristics of their partner’s households, not their natal households (Ji et al. 2013). If wealth has no obvious influence on male reproductive success, but does help daughters reproduce, this might predict daughter-biased investment (Holden et al. 2003). But then competition between sisters can push their mother’s investment toward favoring sons (Johnstone and Cant 2010). Therefore, kin selected benefits and the costs of sibling competition may be pushing sex-biased investment in different directions in the Mosuo.

Both demographic and gift giving data show that mothers help both their adult daughters and their adult sons, but in different ways. Mothers help daughters to breed earlier and enhance the survival of their daughters’ children. This grandmother effect has been found in many other populations with different social organizations (Sear and Mace 2008), although it does not concur with the only other study we are aware of where grandmother effects have been examined in a matrilineal group, the Chewa of Malawi, where grandmothers did not improve child survival (Sear 2008) (although it should be noted the Chewa have matrilocal not duolocal residence). The demographic and gift data show that Mosuo women help both sons and daughters but only matrilineal grandchildren.

The benefits of Mosuo mothers on their adult son’s survival or reproductive success are not as great as they appear to be in killer whales (Foster et al. 2012; Brent et al. 2015). However, Mosuo grandmothers play a role in keeping their adult sons in the natal household, where they continue to support them. The effect of mother’s death is not large, but increases the chances of males dispersing, which should be considered to be an inherent characteristic of this system. Following the death of their mothers, Mosuo men experience a 1.4-fold increase in hazard of dispersal, whereas women do not show any increased risk of dispersal. There is no sex difference in the obligations of sons and daughters to their mother in Mosuo. If dispersal at death of mother reflects termination of responsibility for offspring transfers to parents, we should expect similar effects of maternal death on dispersal of daughters and sons. But the results show that maternal death was followed by increased dispersal of males but not females, suggesting that maternal death reflects the cessation of benefits that a male received from living with his mother. Mothers may help adult sons stay in the natal household to enable them to mate more freely and/or do relatively less work than if they moved into their wife’s household. In matrilineal systems in Malawi, where males move into the wives’ household (uxorilocal), women praise and expect hard work from males, which can earn a husband more rights within the family over time, whereas laziness is cited as reason for ridicule and divorce (Phiri 1983; Kishindo 2011). Matrilocal (or uxorilocal) societies are not present in southwestern China, but in duolocal households, we have previously shown that men do relatively less work than women or than married neolocal Han men in the study site (Wu et al. 2013).

However, the survival of the mother is not associated with a significant bringing forward of their son’s age at first birth (although the trend is in a favorable direction); nor with the survival of their son’s children; nor do they give their son’s children monetary gifts.

Siblings we estimate, on average, have at least a 20% chance of not sharing a father. We found that multiple sisters delayed first birth and increased the probabilities of dispersal of Mosuo women, which are consistent with previous studies that Mosuo sisters are in competition (Ji et al. 2013). Competition between half siblings should be tenser than between full siblings (Hamilton 1964b; Ji et al. 2014). Mosuo women are in more severe competition within the household than men; thus, Mosuo women may be more likely than men to disperse from the natal household when they get opportunities during modernization (such as increased chances of finding a job or a husband outside of the area). Mosuo women born after 1970 experience a higher hazard ratio of dispersal than do older women, whereas men do not show the same pattern (Table 1), showing some support for the sibling competition hypothesis. Sisters do not drive brothers out but give fewer monetary gifts to brothers than to sisters. This may be due to reciprocal altruism between sisters, because they work in close collaboration on both farming and domestic chores, whereas brothers do relatively less agricultural labor or household chores (Wu et al. 2013). Being members of the same working group predicted gift giving in Saami herders (Thomas et al. 2015), and hunter-gatherers gave more gifts to campmates than to those living in other camps (Apicella et al. 2012). Furthermore gifts to female coresidents may be more likely to stay within the communal household and thus relieve household stress and effectively benefit the donor directly, whereas gifts to males may end up being given to their spouse/girlfriend’s or friends’ households. Duolocal groups in the area appear less willing to cooperate and give gifts to those in other households than did patrilocal groups in the area, perhaps due to low rates of dispersal being associated with more competition within villages (Wu et al. 2015).

Our results are in line with historical ethnographic descriptions of Mosuo that somewhat neglect the role of the biological father and paternal grandmother (Cai 2001; Shih 2009). A recent study (Mattison et al. 2014) found evidence for positive effects of coresident fathers on their children’s education and first birth age in Mosuo, but their results based on cross-sectional data are from an area with higher neolocality and might not apply to duolocal households (where fathers live elsewhere). Our gift game results concur with Mattison et al.’s (2014) self- and partner-reports of fathering activities, and showing that currently Mosuo fathers do some direct investment in their own children. This may reflect an increase in paternal investment in Mosuo as a result of modernization and increased exposure to mainstream Chinese culture. Recently, this area has become a local tourist hotspot, and the economic opportunities this and other developments provide, combined with increased contact and intermarriage with non-Mosuo groups, is causing the system to change (Mattison 2010; Mattison et al. 2014; Ji et al. 2016). These changes include not just a reduction in the size of matrilineal households but also an increase in neolocal nuclear households, and the facilitated dispersal out of the area for young people. Ji et al. (2016) predict that once this process starts it could ultimately lead to the loss of the duolocal residence system in favor of neolocality. However, it should be noted that Mosuo fathers were less likely than mothers to give gifts to their own children. Mosuo men were more likely than women to give gifts to people who were not their close kin, suggesting that they look more outside the household than women. Both Mosuo men and women gave more gifts to distant kin/non-kin of the same sex as themselves. This may reflect that reciprocal altruism outside of the natal household mainly happens among members of the same sex in daily life.

Using both demographic data and a gift decision, we show how kin selection and local competition interact in a rare duolocal system in which female kin breed communally. Our results partly supported Johnstone and Cant’s (2010) predictions, as Mosuo mothers tend to increase their inclusive fitness by keeping their sons in the maternal household. But they also show positive effects in helping their daughters reproduce, which shows support to Holden et al.’s (2003) hypothesis. Help for males may be aimed at helping their mating effort, whereas help for females is geared toward raising children and grandchildren. Mothers can benefit from investing in the mating effort of sons, as multiple partners increase reproductive success for men. Further study could examine how exactly mothers or household resources affect the mating effort, partner number, and living offspring number of males (but data from older generations would be more suitable for this as Mosuo were a natural fertility and less restricted population before the 1980s).

We do not know how this unusual duolocal residence system arose historically, but if duolocality is a case of delaying male dispersal until after the mother dies, it suggests duolocality may have arisen from a matrilocal ancestral state. Overall results suggest a key role for the matrilineal grandmother in a duolocal matrilineal society, which should be considered important for understanding this and other matrilineal systems within the framework of evolutionary theory.

## SUPPLEMENTARY MATERIAL

Supplementary material can be found at http://www.beheco.oxfordjournals.org/

## FUNDING

This work was supported by the China Postdoctoral Science Foundation (grant nos. 2013M541036 and 2014T70122 [Q.-Q.H.]), the ERC (grant 249347 on the Evolution of cultural norms in real world settings [J.-J.W. and R.M.]), the NSFC (General Program grant NSFC31270439, 11471311, and 31470453 [T.J. and Y.T.]), and an International Partnership grant from the British Academy (all authors).

## Supplementary Material

Supplementary Data
