# Supplementary material for: Not leaving home: grandmothers and male dispersal in a duolocal human society
Source: Behav Ecol. 2016 Apr 6;27(5):1343–52. doi: 10.1093/beheco/arw053 (PMC5027622; doi:10.1093/beheco/arw053)
Supplement: Supplementary Data [file supp_27_5_1343__index.html]

Not leaving home: grandmothers and male dispersal in a duolocal human society — Not leaving home: grandmothers and male dispersal in a duolocal human society — Not leaving home: grandmothers and male dispersal in a duolocal human society — Supplementary Data 

# Not leaving home: grandmothers and male dispersal in a duolocal human society

## Supplementary Data

Data files

- Supplementary Data - Supplementary Data
