# Supplementary material for: Not leaving home: grandmothers and male dispersal in a duolocal human society
Source: Behav Ecol. 2016 Apr 6;27(5):1343–52. doi: 10.1093/beheco/arw053 (PMC5027622; doi:10.1093/beheco/arw053)
Supplement: Supplementary Data [file supp_arw053_HEQQ_Supporting_Information_snd.doc]

Supporting Information for “Not leaving home: grandmothers and male dispersal in a duolocal human society”

Table of content

1. Partners’ sibling effect on which household males disperse into
2. Paternal grandmother effect on child survival
3. Mother death effect on survival of adult men
4. Partner number and sibling relatedness
   1. Distribution of mother’s reproductive partner number from sibling paternity reports
   2. Full, half and father-unknown sibling of the same mother
5. Sex differences in gift decision (chi-square test)
6. Partners’ sibling effect on which household a male disperses to

The sample includes 398 men with complete information, born and living in this population in their lifetime, who dispersed from their natal household to live with their partners. There are 278 of them dispersed to a neolocal household, and 120 into their partners’ natal household.  The more siblings a man’s partner has, the less likely he will disperse into her maternal household (Table S1).

**Table S1. Results of logistic regressions of the effects of partners’ sibling on which household males disperse into (*n* = 398 dispersed males). We used logistic regression, with residence type after dispersal (matrilocal = 1, neolocal = 0) as dependant variable. The predictors used in the model are number of partner's adult sisters, number of partner's adult brothers, and birth cohort. Significant effects are indicated in bold.**

| Residence type | Estimate (SE) | Odds ratio | *P* |
| --- | --- | --- | --- |
| Partner's adult sister number | **-0.21 (0.097)** | **0.811** | **0.03** |
| Partner's adult brother number | **-0.193 (0.086)** | **0.824** | **0.025** |
| Birth cohort (< 1940 as reference category) | | | |
| 1940s | 0.511 (0.397) | 1.666 | 0.198 |
| 1950s | 0.31 (0.398) | 1.364 | 0.436 |
| 1960s | -0.082 (0.374) | 0.921 | 0.826 |
| 1970s | -0.574 (0.409) | 0.563 | 0.161 |
| Constant | -0.242 (0.325) | 0.785 |  |

1. Paternal grandmother effect on child survival

Table S2 shows the results of the event history analysis of children’s survival during first 6 years, with paternal age controlled. We found no measurable effect of paternal grandmothers’ death on the survival of their grandchildren during first 6 years, with paternal age controlled (Table S2). There is a large decline of sample size because of missing in the information of paternal grandmothers, also suggesting the little importance of patri-line kin.

**Table S2. Results of event history analysis of kin effects on child survival during first 6 years (*n* = 17,473 person years for 1,531 males and 1,506 females; 28 events for males and 31 for females).** We used relogit regression for event history analysis (King and Zeng 2001a; King and Zeng 2001b), with mortality in the first 6 years of child (dead = 1, alive = 0) as dependant variable. The predictors used in the model are patrilineal grandmother dead as time-varying variables, sex, birth cohort, and paternal age cohort as time-unvarying variables. Significant effects are indicated in bold.

| Mortality in the first 6 years | Estimate (SE) | Hazard ratio | *P* |
| --- | --- | --- | --- |
| Sex (male as reference category) | | | |
| female | 0.111 (0.263) | 1.117 | 0.673 |
| Patri-grandmother (alive as reference category) | | | |
| dead | -0.423 (0. 389) | 0.655 | 0.277 |
| Father's age cohort at birth (<21 as reference category) | | | |
| 21-25 | -0.666 (0.602) | 0.514 | 0.269 |
| 26-30 | -0.662 (0.585) | 0.516 | 0.258 |
| 31-35 | -0.766 (0.625) | 0.465 | 0.221 |
| 36+ | -0.48 (0.633) | 0.618 | 0.448 |
| Birth cohort (< 1950 as reference category) | | | |
| 1950s | -2.162 (1.247) | 0.115 | 0.083 |
| 1960s | **-2.854 (1.175)** | **0.058** | **0.015** |
| 1970s | **-2.91 (1.16)** | **0.054** | **0.012** |
| 1980s | **-3.05 (1.146)** | **0.047** | **0.017** |
| 1990-2012 | **-4.086 (1.159)** | **0.017** | **<0.001** |
| Constant | -0.325 (1.132) | 0.723 | 0.774 |

1. Mothers’ deaths do not affect survival of their adult sons (Table S3).

**Table S3. Results of event history analysis of mother’s death effect on adult Mosuo men’s survival (*n* = 46,338 person years for 2,042 adult men aged 15 and over, events of mortality = 162).** We used complementary log-log regression for event history analysis, with mortality of adult men (dead = 1, alive = 0) as dependant variable. The predictors used in the model are mother dead as time-varying variables, birth cohort as time-unvarying variables. Significant effects are indicated in bold.

| Death of adult Mosuo men | Estimate (SE) | Hazard ratio | *P* |
| --- | --- | --- | --- |
| Mother (alive as reference category) | | | |
| dead | 0.242 (0.207) | 1.274 | 0.242 |
| Birth cohort (<1950 as reference category) | | | |
| 1950s | -0.436 (0.239) | 0.647 | 0.068 |
| 1960s | -0.457 (0.261) | 0.633 | 0.08 |
| 1970s | **-0.721 (0.343)** | **0.487** | **0.036** |
| 1980s | -0.755 (0.472) | 0.47 | 0.109 |
| 1990-1997 | -1.022 (1.035) | 0.36 | 0.323 |
| Constant | **-7.507 (0.373)** | **0.001** | **<0.001** |

1. Partner number and sibling relatedness

4.1. Distribution of mother’s reproductive partner number from sibling paternity reports

23.3% of mothers had more than one reproductive partner in their life time (*n* = 288 mothers).


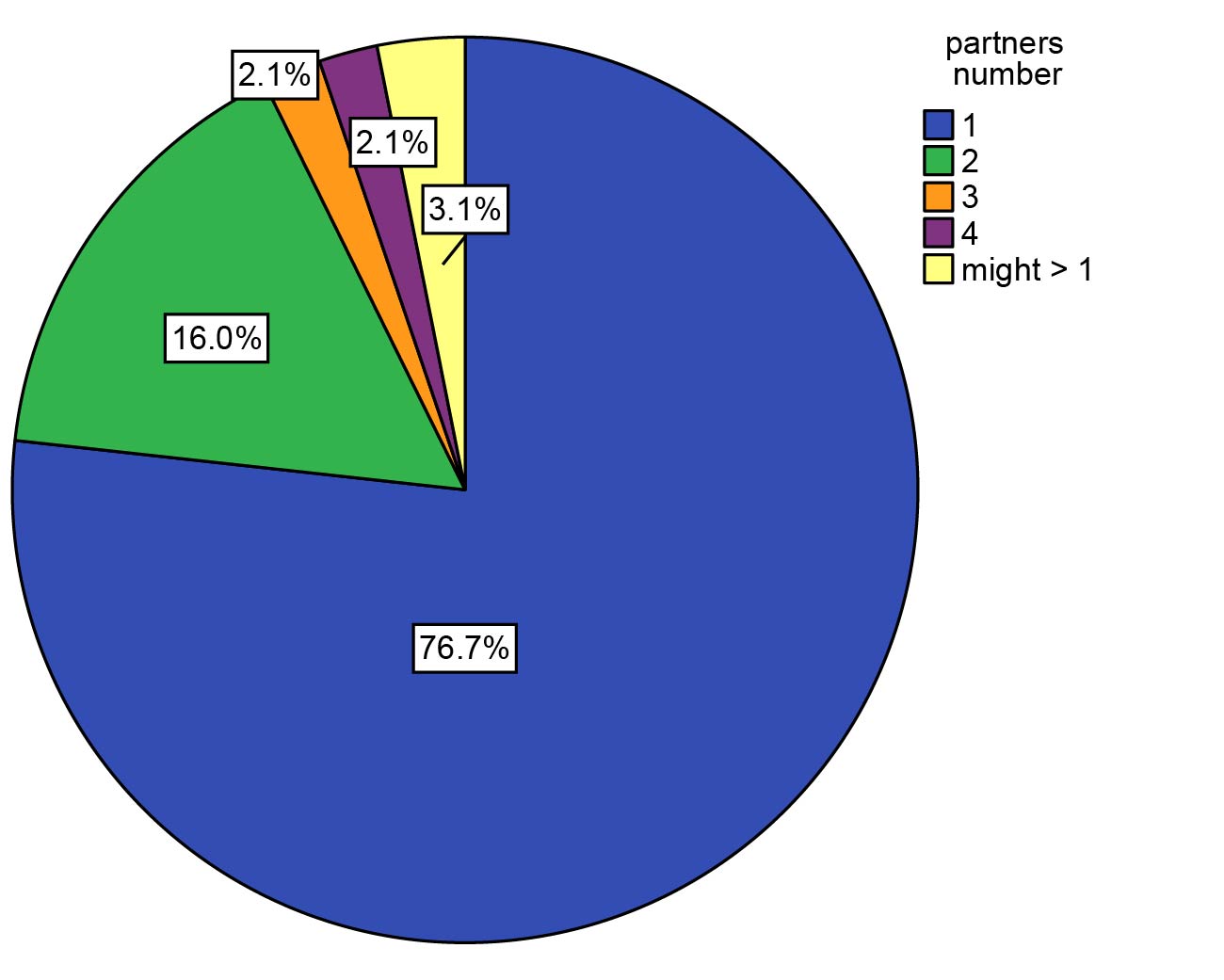


**Figure S1. Distribution of mother’s reproductive partner number from sibling paternity reports (*n* = 288 mothers).** 20.2 % of mothers had more than one reproductive partner during their life time, and an additional 3.1 % of them might have more than one as paternity of at least one sibling is unknown, mean of 1.26 partners per mother, range 1 - 4, SD = 0.602.

4.2. Full, half and father-unknown sibling of the same mother

Siblings are less related to each other on average than their mother is related to them (Table S4).

**Table S4. Full, half and father-unknown sibling of the same mother, mean and SD (*n* = 1,357 offspring of 288 mothers).**

|  | N | Mean siblings number of same mother (SD) | Mean full siblings number (SD) | Mean siblings number of different father (SD) | Mean siblings number of father who might be different (SD) |
| --- | --- | --- | --- | --- | --- |
| Female | 695 | 4.55 (2.077) | 4.03 (2.44) | 0.42 (1.088) | 0.1 (0.469) |
| Male | 646 | 4.77 (2.017) | 4.29 (2.389) | 0.37 (1.022) | 0.1 (0.559) |
| Total | 1,357 | 4.66 (2.049) | 4.15 (2.415) | 0.40 (1.059) | 0.1 (0.522) |

1. Sex differences in gift decision (chi-square test)

Most gifts were given to close kin, with the proportion being higher for women than men. Mosuo women gave similar gifts to distant kin and kin’s spouses with men. They rarely gave gifts to spouses but gave a bit more gifts to spouse’s kin than men. Moreover, Mosuo men were more likely than women to give gifts to other non-kin (chi-square = 34.843, *P* <0.001).


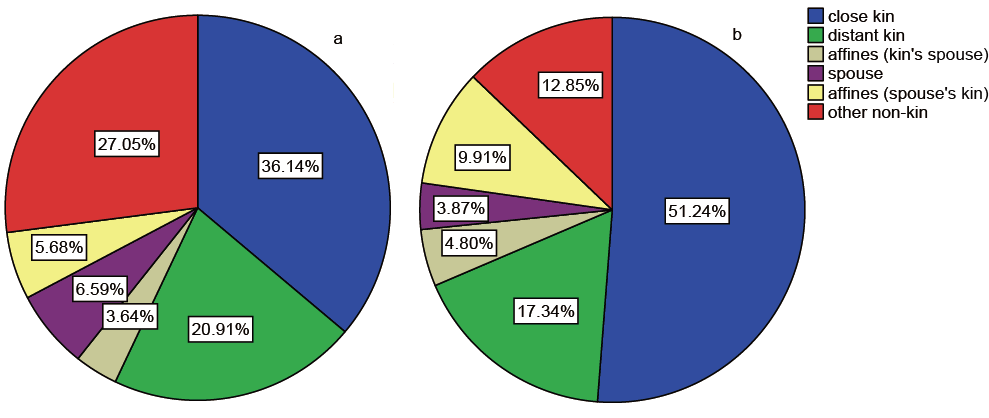


Figure S2. **Distribution of gifts given to different receivers (a) Mosuo male givers aged 18 and over (gifts given to *n* = 440 receivers by 246 male givers), (b) Mosuo female givers aged 18 and over (gifts given to *n* = 646 receivers by 321 female givers).**

We compared whether Mosuo men gave more gifts to mother than women. When the total number of gifts was taken into accounts, Mosuo women were just a little bit more likely than men to give gifts to mother (11.6% for women and 8.6% for men). Women were more likely than men to give gifts to their own children/grandchildren, while they were less likely to give gifts to people out of these close kin (close kin here mean parents, siblings, children, grandchildren and nephews and nieces). Mosuo women gave a bit less gifts to their spouses (6.4% for men and 3.9% for women, no significant differences with chi-square = 3.506, *P* = 0.061).

**Table S5. The sex differences of gifts decision for Mosuo (results of chi-square test, with significant sex differences indicated in bold).**

| Receiver | Male giver | Female giver | Chi-square | *P* |
| --- | --- | --- | --- | --- |
| Father | 10 | 15 | 0.003 | 0.958 |
| Mother | 38 | 75 | 2.482 | 0.115 |
| Brother | 19 | 42 | 2.354 | 0.125 |
| Sister | 35 | 63 | 1.03 | 0.31 |
| Son | 15 | 46 | **6.801** | **0.009** |
| Daughter | 20 | 50 | **4.429** | **0.035** |
| Brother's children | 3 | 4 | n/a ^a^ | 1 ^a^ |
| Sister's children | 17 | 22 | 0.159 | 0.741 |
| Son's children | 0 | 1 | n/a | n/a |
| Daughter's children | 2 | 13 | 4.663 | **0.031** |
| Spouse | 29 | 25 | **4.101** | **0.043** |
| Other | 252 | 290 | 16.048 | **<0.001** |
| Total | 440 | 646 |  |  |

^a^ We used Fisher’s exact test here as the expected values in 2 of the cells of the contingency table are below 5.

n/a, not applicable

References

King G, Zeng L. 2001a. Logistic regression in rare events data. Polit Anal 9:137–163.

King G, Zeng L. 2001b. Explaining rare events in international relations. Int Organ 55:693–715.
